# Benefits of Hemicraniectomy Seen Many Years After Malignant Stroke in a Young Patient

**DOI:** 10.3389/fneur.2012.00123

**Published:** 2012-08-02

**Authors:** Anupam Mittal, Geetanjli Mittal, Arthur Partikian, David Liebeskind, Nerses Sanossian

**Affiliations:** ^1^University of CaliforniaIrvine, CA, USA; ^2^Arrowhead Regional Medical CenterColton, CA, USA; ^3^Department of Neurology, University of Southern CaliforniaLos Angeles, CA, USA; ^4^UCLA Stroke CenterLos Angeles, CA, USA

**Keywords:** stroke, middle cerebral artery, hemicraniectomy

## Abstract

The benefits of hemicraniectomy for malignant middle cerebral artery (MCA) stroke may not be apparent in the 3- to 6-months in which final outcomes are assessed in research studies. We present the case of a 15-year-old who underwent hemicraniectomy for malignant MCA stroke and was significantly disabled 3 and 6 months after event. Over the long-term she was able to graduate from university, play tennis, and live an independent life. Although functional independence with only minor disability is relatively rare in adult hemicraniectomy patients, this outcome may be more easily achieved in children during a longer period of follow-up.

## Introduction

Stroke is common in adults, and represents as the leading cause of long-term disability. Because stroke is not common in children, it often goes unrecognized, which leads to delayed diagnosis and fewer acute treatment options (Srinivasan et al., 2009). The mortality and morbidity after infarction of the entire middle cerebral artery (MCA) territory approaches 80% and decompressive hemicraniectomy has been shown to preserve life and decrease severe disability in adults (Juttler et al., 2007). Outcomes in the studies of decompressive hemicraniectomy in all clinical studies were measured at 6 months post-stroke.

The role of hemicraniectomy in pediatric malignant MCA infarction has been explored through case reports (Ramaswamy et al., 2008; Farooq et al., 2009), yet there are no controlled studies. Outcomes in pediatric cases of hemicraniectomy may be vastly different than those in adults. Little is known of the very long-term outcomes many years after such an event. This is an important point because of a recent survey among high school students about preference to undergo decompressive hemicraniectomy in the setting of malignant MCA stroke (Nakagawa et al., 2010). This survey used adult outcome data and found that some high school students would not want to undergo this procedure; it did not take into consideration the unique potential of a pediatric brain for recovery.

We present the case of a 15-year-old girl with malignant right MCA infarction who underwent hemicraniectomy and had a satisfactory outcome at 3 and 6 months. She then went on to have an excellent 5-year outcome, which highlights the potential for recovery in pediatric cases of malignant MCA stroke.

## Case Report

**Figure 1:**
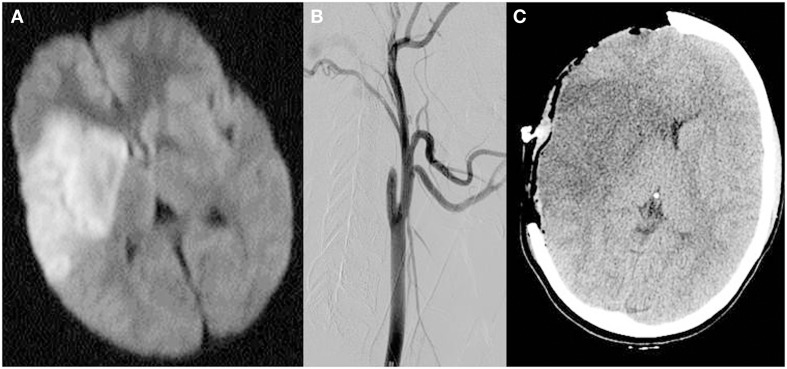
**MRI diffusion-weighted images demonstrating restricted diffusion in the right middle cerebral artery territory including the basal ganglia (A), catheter angiogram demonstrating occlusion of the internal carotid artery distal to the bifurcation (B) and non-contrast head CT scan performed post-hemicraniectomy demonstrating large area of hypodensity in the right middle cerebral territory with mass effect (C)**.

A 15-year-old girl was noted to have sudden onset of headache, confusion, and left sided weakness. A CT scan did not show intracranial hemorrhage and tissue plasminogen activator (TPA) was then administered less than 4 h from symptom onset. The patient was then transferred to a tertiary stroke center where an MRI/MRA demonstrated MCA stroke secondary to presumed right internal carotid artery dissection (Figure 1). The patient was intubated and treated in the pediatric intensive care unit (PICU). Follow-up imaging revealed increasing edema, mass effect, and malignant MCA infarction. A hemicraniectomy was performed less than 20 h after the onset of symptoms (Figure 1C). The patient remained intubated in the PICU for 10 days. After extubation, the neurological examination revealed dense left hemiparesis, hemineglect, and homonymous hemianopia. The patient was transferred to a neurologic rehabilitation ward, received cranioplasty 3 weeks following stroke and was then transferred to inpatient rehabilitation for 3 months. Upon discharge from inpatient rehabilitation the patient was not independent of her activities in daily living, requiring assistance with practically all activities of daily living and was graded as 4 on the modified Rankin scale. Outpatient rehabilitation continued through the next 5 years to this date.

The patient is currently enrolled at university with no cognitive disability and will be graduating this summer. She is functionally independent, able to perform all activities of daily living. She suffers from minimal dysarthria and severe left upper and lower extremity hemiparesis. She is able to walk unassisted with an ankle-foot orthotic. She participates in exercise activities such as tennis. She has obtained her driver’s license and has applied to a master’s program in speech pathology.

## Conclusion

This case report highlights the potential for excellent long-term outcomes in children with malignant MCA infarction and supports findings of earlier studies. Randomized studies of hemicraniectomy are not needed in children; data in adults have demonstrated clear efficacy with trends toward better outcome when younger and healthier. Rather, more long-term observational studies in children are needed. The level of disability at 3 months (mRS 4) described in this case would have been designated a poor outcome in clinical trials which dichotomized mRS into good (mRS 0–3) and poor outcome (mRS 4–6); however the patient’s 5-year outcome would have been categorized as good using the Rankin score of 2 using the focused assessment tool (Saver et al., 2010). This type of improvement in functional status from 3 or 6 months to 5 years is typically not reported in the adult stroke studies (Sulter et al., 1999), and might be attributable to greater mechanisms of plasticity in the younger brain.

Published studies of quality of life in adults have demonstrated that most patients and their relatives would again give consent to hemicraniectomy (Benejam et al., 2009). Clinicians should consider that very long-term outcomes are unknown and may be better than expected when discussing risks and benefits of early hemicraniectomy in pediatric malignant stroke (Smith et al., 2011). We believe that early hemicraniectomy should be considered in all cases of malignant pediatric stroke and that a registry is needed to document functional outcomes over very long periods of observation. Functional independence with only minor disability are relatively rare in adult hemicraniectomy patients, yet may be more easily achieved in children.

## Author Contribution

Study concept and design: Mittal, Mittal, Sanossian. Acquisition of data: Sanossian and Liebeskind. Analysis and interpretation of data: Mittal, Mittal, Partikian, Liebeskind, Sanossian. Drafting of the manuscript: Mittal, Mittal, Partikian, Liebeskind, Sanossian. Critical revision of the manuscript for important intellectual content: Partikian, Liebeskind, Sanossian. Obtained funding: N/A Statistical analysis: N/A. Administrative, technical, and material support: Liebeskind and Sanossian. Study supervision: Sanossian.

## Conflict of Interest Statement

Mr. Mittal has nothing to disclose. Dr. Mittal has nothing to disclose. Dr. Partikian has nothing to disclose. Dr. Liebeskind is supported by NIH (NINDS; K23NS054084 and P50NS044378). Dr. Sanossian is supported by a grant from Joachim Splichal and the Roxana Todd Hodges Foundation.
